# Impact of comorbidity burden and skin integrity on clinical presentation and outcomes in *Streptococcus dysgalactiae* subspecies *equisimilis* bacteremia

**DOI:** 10.1007/s10096-026-05450-3

**Published:** 2026-03-03

**Authors:** Elviira Haapaniemi, Viivi Nevanlinna, Janne Aittoniemi, Reetta Huttunen, Tiina Luukkaala, Sari Rantala

**Affiliations:** 1 Department of Internal Medicine, Tampere University Hospital, Elämänaukio 2, Tampere, 33520 Finland; 2 Faculty of Medicine and Health Technology, Tampere University, Tampere, Finland; 3 Fimlab Laboratories, Tampere, Finland; 4 Research, Development and Innovation Center, Tampere University Hospital, Tampere, Finland; 5 Health Science Faculty of Social Science, Tampere University, Tampere, Finland

**Keywords:** Streptococcus dysgalactiae subspecies equisimilis, Bacteremia, Comorbidity, Charlson Comorbidity Index, Mortality

## Abstract

**Purpose:**

The purpose of this study was to evaluate the impact of comorbidity burden on clinical presentation and mortality in *Streptococcus dysgalactiae* subspecies *equisimilis* (SDSE) bacteremia, and to assess the association between disruption of the cutaneous barrier and clinical manifestation.

**Methods:**

We conducted a prospective study of 159 episodes of SDSE bacteremia in 157 adult patients in Finland between 2015 and 2019. Comorbidity burden was assessed using the Charlson Comorbidity Index (CCI). 30-day mortality and clinical manifestation of SDSE bacteremia were analyzed in relation to comorbidity burden. Disruption of the cutaneous barrier was examined in relation to the presenting clinical manifestation.

**Results:**

Higher comorbidity burden was associated with mortality; a CCI ≥ 4 increased the risk of death fourfold (*p* = 0.035). Cerebral infarction, chronic pulmonary disease—particularly chronic obstructive pulmonary disease—and leukemia were independently associated with mortality. The 30-day mortality was 6%. Cellulitis was more common among patients with a lower comorbidity burden, whereas bacteremia without a defined focus predominated in patients with higher comorbidity burden. Disruption of the cutaneous barrier was associated with skin and soft tissue infections, while endocarditis, bone and joint infections, and bacteremia without a defined focus were more frequent among patients with intact skin.

**Conclusion:**

Comorbidity burden and skin integrity appear to be relevant factors in determining the clinical presentation and prognosis of SDSE bacteremia. These findings suggest that comorbidity assessment and skin evaluation may be useful for understanding the clinical presentation and prognosis of SDSE bacteremia.

## Introduction

*Streptococcus dysgalactiae* subspecies *equisimilis* (SDSE) is a β-hemolytic streptococcus belonging primarily to Lancefield group C or G [1, 2]. SDSE is part of the normal human flora, commonly colonizing the skin, pharynx, gastrointestinal, and genitourinary tracts [3, 4]. The incidence of infections caused by SDSE is increasing and in several countries it has become the most common β-hemolytic streptococcal species causing invasive infections [5–9]. The clinical spectrum of SDSE bacteremia is largely comparable to that of *Streptococcus pyogenes*, ranging from cellulitis to life-threatening necrotizing fasciitis and septic shock or streptococcal toxic shock syndrome (STSS) [3, 10].

Patients with SDSE bacteremia are older and have more comorbidities than those with group A streptococcal bacteremia [6, 11, 12]. Some of the most common comorbidities are cardiovascular disease, diabetes, and malignancies, the majority of patients carrying several underlying diseases [3, 13, 14]. SDSE infections are associated with a higher Charlson Comorbidity Index (CCI) compared with group A and B streptococcal infections, but the association between CCI and mortality in SDSE bacteremia has not been fully examined [15–17]. There appears to be only one study linking higher CCI to increased mortality in invasive group C or G streptococcal infections [15]. The mortality of SDSE bacteremia has been reported to range from 8 to 20% [16, 18–20].

The majority of SDSE bacteremias present with a skin and soft tissue infection [19, 21]. The disruption of the cutaneous barrier has been reported to be a significant predisposing factor in group A, but also in group G β-hemolytic streptococcal bacteremias [5]. In elderly patients with SDSE bacteremia, the primary focus of infection is often unknown [7, 21]. Pneumonia appears to occur more frequently in older patients, whereas septic arthritis and noncutaneous abscesses are more commonly observed in younger individuals [7, 21].

We conducted a prospective study to assess the significance of comorbidities – primarily measured by the Charlson Comorbidity Index (CCI) – on mortality in SDSE bacteremia. Furthermore, we aimed to evaluate whether the extent of comorbidities and the integrity of the skin were associated with the clinical manifestation of the infection in these patients.

## Methods

This prospective study was conducted in Finland in Pirkanmaa Hospital District (HD) from November 2015 to November 2019. We included adult patients with at least one *Streptococcus dysgalactiae* subspecies *equisimilis* (SDSE)-positive blood culture hospitalized either at Tampere University Hospital (TAUH) or Hatanpää City Hospital during the study period. TAUH is the second largest tertiary care unit in Finland, serving approximately 535 000 residents in the Pirkanmaa HD. Hatanpää City Hospital was integrated into TAUH at the beginning of 2017.

Throughout the study period, all SDSE-positive blood cultures were reported by a clinical microbiologist (J.A., T.S. or I.J.) to an infectious disease specialist (S.R.). Additionally, the infectious disease specialist reviewed SDSE bacteremias from the national register for hospital infections and antimicrobial drug use. This register documents all positive blood culture findings in Finland. Blood samples were analyzed at Fimlab laboratories. Three cases of *Streptococcus canis* were excluded. Two patients declined to participate. Both monomicrobial and polymicrobial episodes of SDSE bacteremia were included. Among the 159 episodes, six were polymicrobial with additional organisms including Gram-negative rods, *Staphylococcus aureus* or *Enterococcus*. The final study population consisted of 159 SDSE episodes in 157 patients. The study was approved by the Regional Ethics Committee of Tampere University Hospital.

Until October 2017, blood samples were collected into BacT/Alert Aerobic (FA Plus) and Anaerobic (FN Plus) blood culture bottles and incubated in the BacT/Alert 3D microbial detection system (bioMérieux). From November 2017 onwards, samples were collected using BD BACTEC Plus Aerobic/F and Lytic/10 Anaerobic/F vials and incubated in the BD BACTEC FX system (Becton Dickinson). SDSE identification was initially based on β-hemolysis and the presence of large colonies on blood agar plates. Until February 2017, Lancefield group identification was performed using latex bead agglutination (PathoDxtra Strep Grouping Kit; Thermo Fisher Scientific), and species identification was confirmed using API 20 Strep (bioMérieux) or matrix-assisted laser desorption/ionization time-of-flight (MALDI-TOF) mass spectrometry (VITEK MS; bioMérieux). Since March 2017, MALDI-TOF has served as the primary identification method. MALDI-TOF results reporting *S. dysgalactiae* subsp. *dysgalactiae/equisimilis* were interpreted as *S. dysgalactiae* subsp. *equisimilis* based on its association with human infections.

All patients were interviewed, medical histories reviewed, and the condition of the skin evaluated by an infectious disease specialist (S.R.). Informed consent was obtained from each patient. If a patient was unable to give consent due to an impaired clinical condition, a first-degree relative was contacted.

The Charlson Comorbidity Index (CCI) is used to evaluate the comorbidity burden of an individual [22]. The CCI assigns one point each for history of myocardial infarction, congestive heart failure, peripheral vascular disease, cerebrovascular disease, dementia, chronic pulmonary disease, connective tissue disease, peptic ulcer disease, mild liver disease, and diabetes mellitus (DM) without complications. For hemiplegia, moderate to severe renal disease, DM with end-organ damage, tumor without metastases, leukemia, lymphoma, and myeloma two points are assigned. For moderate or severe liver disease three points are assigned and six points are assigned for metastatic solid tumor or acquired immunodeficiency syndrome (AIDS). CCI was calculated for each patient, using information obtained through interviews and medical records. Modifications to the standard CCI were made as follows: myocardial infarction was replaced by coronary artery disease. Diabetes with end-organ damage referred to diabetes-related renal disease. Ulcer disease and hemiplegia were excluded. Liver disease was assigned one point, as its severity was unknown. No patients with AIDS were present in the study population. Age was not included in the CCI but was treated as a separate variable in the analyses. Analyses involving the CCI were adjusted for age. We chose to use the original CCI score instead of the updated version [23], since it has been predominantly used in previous studies investigating streptococcal infections [15–17].

Bacteremia without a defined focus was classified as a diagnosis of exclusion. Cases were assigned to this category when no clinical, microbiological, or imaging findings indicating a specific infectious focus were identified during the initial diagnostic work-up. At a minimum, this work-up included clinical examination, routine laboratory testing, blood cultures, and targeted imaging or additional investigations at the discretion of the treating physician based on clinical presentation. Endocarditis was defined as the presence of positive blood culture, echocardiographic evidence of valvular vegetation, a clinical presentation consistent with endocarditis, and a diagnosis made by the treating clinician.

Obesity was defined as body mass index > 30 kg/m^2^. Alcohol abuse was defined as a known social or medical problem related to alcohol or consumption exceeding the Finnish recommendations (women ≥ 12 alcohol units/week and men ≥ 23 alcohol units/week). Immunosuppressive treatment was defined as systemic glucocorticoid therapy, active cytostatic therapy and/or the use of biological medications.

We performed statistical analyses using IBM SPSS Statistics for Mac (version 29). We analyzed patient demographics in relation to mortality, which was defined as death within 30 days of hospital admission. Age-adjusted Cox proportional hazard regression was used to calculate hazard ratios (HR) and corresponding 95% confidence intervals (CI). Additionally, we assessed the relationship between CCI and the site of infection, and the disruption of the cutaneous barrier and the site of infection. Logistic regression was used to calculate odds ratios (OR) and corresponding 95% confidence intervals (CI). P-values < 0.05 were considered statistically significant. The predictive value of CCI for mortality was evaluated using receiver operating characteristic (ROC) curves. An area under the curve (AUC) of 1.0 denotes perfect diagnostic accuracy, whereas an AUC of 0.5 denotes no diagnostic capability [24]. The optimal cutoff point for CCI was identified using the Youden index (sensitivity + specificity − 1).

We used GPT-5.2, produced by OpenAI, to enhance the language and fluency of the manuscript. After using this tool, the content was reviewed and edited as needed and the authors take full responsibility for the content of the published article.

## Results

This four-year prospective study was conducted from November 2015 to November 2019 in Tampere University Hospital and Hatanpää City Hospital in Finland. Among the 159 episodes of SDSE bacteremia involving 157 patients, the 30-day mortality was 6% (9 patients died).

The detailed demographics of the study population in relation to mortality are presented in Table 1. Most of the SDSE patients were ≥ 65 years old and male, but age or sex were not associated with mortality. Cerebral infarction (HR 10.79 [95% CI 2.47–47.10], *p* = 0.002), chronic pulmonary disease (HR 5.31 [95% CI 1.37–20.60], *p* = 0.016), and leukemia (HR 11.49 [95% CI 1.39–95.21], *p* = 0.024) were associated with increased mortality in SDSE bacteremia. In chronic pulmonary disease, the association was driven primarily by chronic obstructive pulmonary disease (COPD) (HR 8.21 [95% CI 1.64–41.25], *p* = 0.011).

**Table 1:** Predisposing factors and effect of disease burden on mortality in *Streptococcus dysgalactiae* subsp. *equisimilis* bacteremia

	Survivors *n* = 150		Non-survivors *n* = 9		Non-survivors vs. survivors		
	*n*	(%)	*n*	(%)	*p* value	HR	(95% CI)
Age, years					0.112		
< 65	45	(30)	0	(0)			
≥ 65	105	(70)	9	(100)			
Gender							
Male	89	(59)	5	(56)		1.00	
Female	61	(41)	4	(44)	0.936	1.06	(0.27–4.19)
CCI							
< 4	105	(70)	3	(33)		1.00	
≥ 4	45	(30)	6	(67)	0.035	4.44	(1.11–17.76)
Median	2	0–9	4	2–8			
Obesity	82	(55)	5	(56)	0.882	1.11	(0.30–4.15)
Alcohol abuse	18	(12)	1	(11)	0.740	1.43	(0.17–12.03)
Cardiovascular disease	61	(41)	6	(67)	0.381	1.94	(0.44–8.59)
Coronary artery disease	31	(21)	5	(56)	0.075	3.49	(0.88–13.75)
Chronic heart failure	47	(31)	4	(44)	0.825	1.17	(0.30–4.64)
Peripheral artery disease	13	(9)	0	(0)		NA	
Neurological disease	14	(9)	3	(33)	0.117	3.15	(0.75–13.26)
Cerebral infarction	3	(2)	3	(33)	0.002	10.79	(2.47–47.10)
Dementia	7	(5)	2	(22)	0.130	3.52	(0.69–17.99)
Chronic pulmonary disease	21	(14)	4	(44)	0.016	5.31	(1.37–20.60)
COPD	5	(3)	2	(22)	0.011	8.21	(1.64–41.25)
Asthma	13	(9)	2	(22)	0.153	3.18	(0.65–15.53)
Rheumatic disease	10	(7)	1	(11)	0.814	1.29	(0.16–10.38)
Chronic liver disease	10	(7)	0	(0)		NA	
Diabetes	60	(40)	4	(44)	0.874	1.11	(0.30–4.15)
End-organ damage	10	(7)	0	(0)		NA	
Chronic kidney disease	20	(13)	1	(11)	0.763	0.73	(0.09–5.84)
Malignancy	41	(27)	4	(44)	0.275	2.08	(0.56–7.77)
Solid tumor	37	(25)	3	(33)	0.562	1.51	(0.38–6.04)
Metastatic	17	(11)	2	(22)	0.177	3.05	(0.61–15.33)
Leukemia	1	(1)	1	(11)	0.024	11.49	(1.39–95.21)
Lymphoma	4	(3)	0	(0)		NA	
Immunosuppressive treatment	22	(15)	1	(11)	0.997	1.00	(0.12–8.25)

The results of age-adjusted Cox proportional hazard regression are shown by hazard ratios (HR) with 95% confidence intervals (CI)

*CCI  Charlson Comorbidity Index

^†^*COPD * chronic obstructive pulmonary disease

^‡^*NA * not applicable

**Fig. 1:**
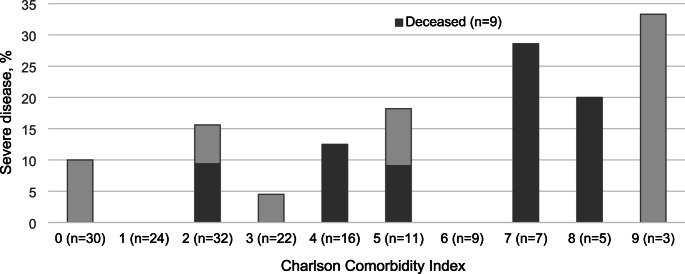
Association between Charlson Comorbidity Index and severe disease (*n* = 17) in SDSE bacteremia (*n* = 159). Severe disease was defined as intensive care unit (ICU) admission and/or death within 30 days of a positive blood culture

A higher comorbidity burden, as represented by CCI, seemed to be an indicator of poor prognosis in SDSE bacteremia (Fig. 1). We used receiver operating characteristic (ROC) analysis to obtain an optimal cutoff value for CCI to predict mortality. ROC analysis demonstrated an AUC of 0.726 (95% CI 0.582–0.869; *p* = 0.002). According to the Youden index, the optimal cutoff was CCI ≥ 4 providing the best discrimination. The cutoff value was significantly associated with 30-day mortality, having 67% sensitivity and 70% specificity. Table 1 shows that CCI ≥ 4 resulted in an over fourfold increased risk of mortality (HR 4.44 [95% CI 1.11–17.76], *p* = 0.035). The median CCI was higher among non-survivors (4, range 2–8) than among survivors (2, range 0–9).

The associations between comorbidity burden measured by CCI and clinical manifestations are shown in Table 2. Cellulitis was significantly associated with lower CCI scores (OR 0.44 [95% CI 0.22–0.90], *p* = 0.024), whereas bacteremia without a defined focus was significantly more frequent in patients with a higher comorbidity burden (OR 6.22 [95% CI 2.06–18.85], *p* < 0.001).

**Table 2:** Clinical manifestations of *Streptococcus dysgalactiae* subsp. *equisimilis* bacteremia in relation to the Charlson Comorbidity Index (CCI)

	CCI < 4 *n* = 108		CCI ≥ 4 *n* = 51		CCI ≥ 4 vs. CCI < 4		
Presenting clinical manifestation	*n*	(%)	*n*	(%)	*p* value	OR	(95% CI)
Skin/soft tissue infection	83	(77)	34	(67)	0.190	0.61	(0.29–1.28)
Cellulitis	81	(75)	29	(57)	0.024	0.44	(0.22–0.90)
Purulent skin infection	17	(16)	14	(28)	0.055	2.25	(0.98–5.15)
Necrotizing fasciitis	2	(2)	2	(4)	0.385	2.45	(0.32–18.62)
Deep abscess	11	(10)	4	(8)	0.728	0.81	(0.24–2.70)
Bursitis	2	(2)	0	(0)		NA	
Bone and joint infection	13	(12)	5	(10)	0.653	0.78	(0.26–2.32)
Osteomyelitis	6	(6)	3	(6)	0.945	1.05	(0.25–4.40)
Spondylitis	5	(5)	3	(6)	0.742	1.28	(0.29–5.60)
Arthritis	10	(9)	2	(4)	0.248	0.40	(0.08–1.90)
Periprosthetic joint infection	5	(5)	1	(2)	0.404	0.40	(0.05–3.50)
Pneumonia	17	(16)	7	(14)	0.656	0.80	(0.31–2.11)
Empyema	1	(1)	0	(0)		NA	
Endocarditis	3	(3)	2	(4)	0.724	1.39	(0.22–8.61)
Aortitis	1	(1)	0	(0)		NA	
Foreign body infection	1	(1)	2	(4)	0.236	4.35	(0.38–49.44)
Puerperal sepsis	4	(4)	0	(0)		NA	
Intra-abdominal infection	2	(2)	0	(0)		NA	
Endophthalmitis	1	(1)	1	(2)	0.586	2.18	(0.13–36.02)
Bacteremia without defined focus	5	(5)	12	(24)	0.001	6.22	(2.06–18.85)

The results of age-adjusted logistic regression are presented by odds ratios (OR) and 95% confidence intervals

*CCI  Charlson Comorbidity Index

^†^*NA * not applicable

The possible portal of entry of the bacteremia through cutaneous disruption (chronic eczema, skin lesion, chronic wound, traumatic wound or oedema) was examined in relation to the clinical manifestations (Table 3). Skin and soft tissue infections were significantly more common in patients with disrupted skin (84%) compared to those without (37%). Specifically, cellulitis (OR 7.22 [95% CI 3.18–16.42], *p* < 0.001) and purulent skin infections (OR 5.04 [95% CI 1.14–22.27], *p* = 0.033) were associated with disrupted skin. Conversely, although the number of patients was considerably low in subgroups other than skin and soft tissue infections, endocarditis (OR 0.06 [95% CI 0.01–0.58], *p* = 0.015) and bacteremia without a defined focus (OR 0.20 [95% CI 0.07–0.57], *p* = 0.002) were statistically associated with intact skin. Bone and joint infections, and particularly arthritis (OR 0.25 [95% CI 0.07–0.82], *p* = 0.022), were also more frequently observed in patients with intact skin.

**Table 3:** Presenting clinical manifestation of *Streptococcus dysgalactiae* subsp. *equisimilis* bacteremia in patients with intact or disrupted skin

	Intact skin *n* = 35		Disruption of cutaneous barrier *n* = 124		Disrupted vs. intact		
Presenting clinical manifestation	*n*	(%)	*n*	(%)	*p* value	OR	(95% CI)
Skin/soft tissue infection	13	(37)	104	(84)	< 0.001	8.80	(3.81–20.31)
Cellulitis	12	(34)	98	(79)	< 0.001	7.22	(3.18–16.42)
Purulent skin infection	2	(6)	29	(23)	0.033	5.04	(1.14–22.27)
Necrotizing fasciitis	0	(0)	4	(3)		NA	
Deep abscess	2	(6)	13	(11)	0.401	1.93	(0.42–9.00.42.00)
Bursitis	1	(3)	1	(1)	0.368	0.28	(0.02–4.54)
Bone and joint infection	8	(23)	10	(8)	0.019	0.30	(0.11–0.82)
Osteomyelitis	3	(9)	5	(4)	0.289	0.45	(0.10–1.98)
Spondylitis	3	(9)	5	(4)	0.289	0.45	(0.10–1.98)
Arthritis	6	(17)	6	(5)	0.022	0.25	(0.07–0.82)
Periprosthetic joint infection	3	(9)	3	(2)	0.113	0.26	(0.05–1.37)
Pneumonia	3	(9)	21	(17)	0.232	2.18	(0.61–7.77)
Empyema	0	(0)	1	(1)		NA	
Endocarditis	4	(11)	1	(1)	0.015	0.06	(0.01–0.58)
Aortitis	1	(3)	0	(0)		NA	
Foreign body infection	1	(3)	2	(2)	0.637	0.56	(0.05–6.33)
Puerperal sepsis	2	(6)	2	(2)	0.199	0.27	(0.04–1.99)
Intra-abdominal infection	1	(3)	1	(1)	0.368	0.28	(0.02–4.54)
Endophthalmitis	2	(6)	0	(0)		NA	
Bacteremia without defined focus	9	(26)	8	(7)	0.002	0.20	(0.07–0.57)

Disruption of the cutaneous barrier includes chronic eczema, skin lesion, chronic wound, traumatic wound and chronic oedema. The results of the logistic regression are presented with odds ratios (OR) and 95% confidence intervals (CI)

*NA  not applicable

## Discussion

A higher comorbidity burden, commonly assessed by the CCI, has been shown to predict poor outcome in invasive β-hemolytic streptococcal infections. Couture-Cossette et al. observed higher mortality with a higher CCI score in invasive group C and G streptococcal infections [15], and Laupland et al. found that in β-hemolytic streptococcal bacteremias a higher CCI predicted poor outcome [16]. Bläckberg et al. examined the association between CCI and mortality in SDSE bacteremia and found no significant association using a cutoff of ≥ 3 [17]. However, the rationale for this cutoff was not specified, and it remains unclear whether higher CCI scores might be associated with mortality. In our study, we aimed to identify the cutoff with the best discriminatory performance within our cohort. More generally, studies applying the CCI should clearly justify their chosen cutoff, as reliance on a single categorical threshold may not fully capture its association with mortality. In our study a higher CCI, reflecting the overall comorbidity burden of an individual, was associated with increased mortality in SDSE bacteremia.

Group C and G streptococcal bacteremias have been associated with both higher CCI scores and mortality than group A and B streptococci [16]. It is noteworthy, that although *Streptococcus pyogenes* typically causes more severe disease, mortality among patients with SDSE may be even higher [15, 16]. Patients with SDSE bacteremia tend to be elderly individuals having a higher burden of comorbidities than those with *Streptococcus pyogenes* [4]. In the present study, we observed that multimorbidity might be an independent risk factor for mortality in SDSE bacteremia, even after adjusting for age. This may explain the possibly even higher mortality among SDSE bacteremia compared to group A and B streptococcal bacteremias.

We observed that cerebral infarction, chronic pulmonary disease, especially COPD, and leukemia were associated with poor outcome in SDSE bacteremia. Due to the small sample sizes for these comorbidities, additional studies are needed to confirm these findings. Although cardiovascular diseases, diabetes, obesity, alcohol abuse and immunosuppression are common underlying conditions, they did not individually associate with mortality. Studies evaluating the impact of specific underlying conditions on mortality in SDSE bacteremia are rare. In retrospective, population-based cohorts from the Pirkanmaa region in southern Finland, Rantala et al. observed that alcoholism, liver disease and having an ultimately or rapidly fatal underlying disease were associated with mortality in β-hemolytic streptococcal bacteremia [18] and Saukkosaari et al. observed that alcoholism and malignancies were associated with severe disease in SDSE bacteremia [25]. Ekelund et al. reported that chronic heart or lung diseases, alcohol abuse or immunodeficiency were associated with poor outcome in invasive β-hemolytic infections [13]. In conclusion, the underlying conditions predisposing to mortality or severe disease are heterogeneous, and their relative importance varies across studies. This supports the conclusion that the overall burden of comorbidities, rather than any single disease, plays a greater role in the development of severe disease and increased mortality in SDSE bacteremia.

As with earlier studies, skin and soft tissue infections were the most common manifestation of SDSE bacteremia. Interestingly, cellulitis was more frequently observed in patients with fewer comorbidities and disrupted skin, whereas bacteremia without a defined focus was more common among those with a higher comorbidity burden but intact skin. It is possible that minor, clinically subtle skin disruptions could predispose frailer patients to SDSE bacteremia, whereas such minimal breaches might not lead to an infection in individuals with fewer comorbidities. Furthermore, in patients with a high burden of comorbidities, the infection focus is more frequently undetected, as these patients may be less likely to undergo extensive diagnostic evaluation, such as computer tomography imaging. This could result in a higher proportion of undefined infection foci.

Our findings suggest that disruption of the cutaneous barrier might influence the site of SDSE bacteremia. Skin and soft tissue infections, particularly cellulitis and purulent skin infections, were markedly more frequent in patients with disrupted skin. In contrast, bone and joint infections, such as arthritis, as well as endocarditis and bacteremia without a defined focus, were more common among patients with intact skin. In light of these findings, it is possible that in these episodes, SDSE bacteremia typically originates from a source other than the skin. All patients were systematically examined by the same infectious diseases specialist (S.R.), representing a major strength of this prospective study. In a prospective study by Kailankangas et al. *Streptococcus pyogenes* was frequently observed in the throat among patients with invasive group A streptococcus infection, which may suggest that the nasopharynx serves as a portal of entry in these patients [26]. In one study including several different bacteria, the most common portal of entry for infective endocarditis was cutaneous, followed by oral/dental and gastrointestinal sources [27].

The strengths of our study were its prospectivity, and that the study population was relatively large. All data were collected comprehensively by the same infectious disease specialist (S.R.), who conducted detailed interviews and individually examined the integrity of each patient’s skin. To ensure a more comprehensive assessment of the patients’ comorbidity burden, we also assessed other clinically relevant conditions in addition to those covered in CCI. There are also some limitations in our study. The CCI is a widely used tool for assessing comorbidity burden in clinical research, but parts of it are outdated and may overestimate the impact of certain diseases, as treatments for conditions such as AIDS and some cancers have improved considerably, leading to a markedly improved prognosis. Because of the limited number of endpoints, confirmation of these results requires replication in other datasets.

## Conclusion

A higher CCI score indicating the comorbidity burden of a patient was associated with higher mortality in SDSE bacteremia. Cerebral infarction, chronic pulmonary disease, especially COPD, and leukemia were independently associated with poor outcome. Cellulitis was more frequently observed in patients with fewer comorbidities, whereas bacteremia without a defined focus was more common among those with a higher comorbidity burden. Skin and soft tissue infections were markedly more frequent in patients with disrupted skin, whereas bone and joint infections, endocarditis and bacteremia without a defined focus were more common among patients with intact skin. This might suggest that in these deep-seated infections, the portal of entry for SDSE bacteremia may originate from a source other than the skin.

## Data Availability

The dataset is not publicly available owing to individual privacy, but it is available from the corresponding author on reasonable request.
